# Live Feeds Used in the Larval Culture of Red Cusk Eel, *Genypterus chilensis*, Carry High Levels of Antimicrobial-Resistant Bacteria and Antibiotic-Resistance Genes (ARGs)

**DOI:** 10.3390/ani10030505

**Published:** 2020-03-18

**Authors:** Luz Hurtado, Claudio D. Miranda, Rodrigo Rojas, Félix A. Godoy, Mark A. Añazco, Jaime Romero

**Affiliations:** 1 Programa Cooperativo de Doctorado en Acuicultura, Departamento de Acuicultura, Universidad Católica del Norte, Coquimbo 1780000, Chile; luzhurtado21@hotmail.com; 2 Laboratorio de Patobiología Acuática, Departamento de Acuicultura, Universidad Católica del Norte, Coquimbo 1780000, Chile; rrojas@ucn.cl; 3 Centro AquaPacífico, Universidad Católica del Norte, Coquimbo 1780000, Chile; 4 Centro i~mar, Universidad de Los Lagos, Puerto Montt 5480000, Chile; felix.godoy@ulagos.cl (F.A.G.); markanazco@gmail.com (M.A.A.); 5 Laboratorio de Biotecnología, Instituto de Nutrición y Tecnología de los Alimentos (INTA), Universidad de Chile, Macul, Santiago 7810000, Chile; jromero@inta.uchile.cl

**Keywords:** resistant bacteria, red cusk eel, *Genypterus chilensis*, florfenicol, vibrios, live feed, rotifer, *Artemia*, *floR*, *fexA*

## Abstract

**Simple Summary:**

The culture of the marine fish red cusk eel *Genypterus chilensis* is currently considered a priority for Chilean aquaculture but low larval survival rates have prompted the need for the continuous use of antibiotics, mainly florfenicol. In this study, the role of live prey (rotifers and the brine shrimp *Artemia franciscana*) used to feed fish larvae as a source of antibacterial-resistant bacteria in a commercial culture of *G. chilensis* was investigated. Samples of live feeds were collected during the larval growth period and their bacterial contents were determined. High levels of potentially opportunistic pathogens, such as *Vibrio* spp., as well as florfenicol-resistant bacteria, were detected. Sixty-five florfenicol-resistant isolates were recovered from these cultures and identified as *Vibrio* (81.5%) and *Pseudoalteromonas* (15.4%), which exhibited a high incidence of co-resistance to the antibiotics streptomycin, oxytetracycline, co-trimoxazole, and kanamycin. The majority of them carried the florfenicol-resistance encoding genes *floR* and *fexA*. The high prevalence of antibiotic-resistant bacteria and the associated genetic elements in live feed administered to reared fish larvae requires the prompt implementation of efficient management strategies to prevent future therapy failures in fish larval cultures and the spread of antibiotic-resistant bacteria to associated aquatic environments.

**Abstract:**

The culture of red cusk eel *Genypterus chilensis* is currently considered a priority for Chilean aquaculture but low larval survival rates have prompted the need for the continuous use of antibacterials. The main aim of this study was to evaluate the role of live feed as a source of antibacterial-resistant bacteria in a commercial culture of *G. chilensis*. Samples of rotifer and *Artemia* cultures used as live feed were collected during the larval growth period and culturable bacterial counts were performed using a spread plate method. Rotifer and *Artemia* cultures exhibited high levels of resistant bacteria (8.03 × 10^4^ to 1.79 × 10^7^ CFU/g and 1.47 × 10^6^ to 3.50 × 10^8^ CFU/g, respectively). Sixty-five florfenicol-resistant isolates were identified as *Vibrio* (81.5%) and *Pseudoalteromonas* (15.4%) using 16S rRNA gene sequence analysis. A high incidence of resistance to streptomycin (93.8%), oxytetracycline (89.2%), co-trimoxazole (84.6%), and kanamycin (73.8%) was exhibited by resistant isolates. A high proportion of isolates (76.9%) carried the florfenicol-resistance encoding genes *floR* and *fexA*, as well as plasmid DNA (75.0%). The high prevalence of multiresistant bacteria in live feed increases the incidence of the resistant microbiota in reared fish larvae, thus proper monitoring and management strategies for live feed cultures appear to be a priority for preventing future therapy failures in fish larval cultures.

## 1. Introduction

The current trend in Chilean marine aquaculture is toward the diversification of cultured aquatic species. The priority is to develop the culture of native marine fish species, in particular, the red cusk eel (*Genypterus chilensis*), which is a Chilean native species of high gastronomic demand and market value. Consequently, it has been identified as a good candidate for production on a commercial scale. Until now, biological studies of the red cusk eel have focused mostly on genetic diversity and population structure [1,2].

The occurrence in reared diseased *G. chilensis* of various opportunistic pathogens, predominantly within the genus *Vibrio,* including *Vibrio anguillarum*, *Vibrio ordalii*, *Vibrio tapetis*, and *Vibrio toranzoniae* species have been reported previously [3,4,5]. Furthermore, the pathogenic species *Tenacibaculum dicentrarchi* was detected in this fish species [6]. However, studies on the sanitary conditions during the culture of red cusk eel, including administered live feed during the larval stage are not available, despite the recognized importance of live feed as an important source of bacterial contaminants in marine fish larval cultures [7,8,9].

Live prey used to feed reared marine larval fish (rotifer *Brachionus plicatilis* and brine shrimp *Artemia franciscana*), as well as intensive rearing conditions, are the main causes for the proliferation of bacteria, which could be responsible for the reduced growth and increased mortality of reared larvae [10,11,12,13]. As was previously noted by Vallés et al. [14], as soon as fish larvae start to capture and ingest live prey, larvae bacterial levels increase exponentially, with *Vibrio* species being the main component of the gut microbiota that are mainly derived from live prey [15,16]. Furthermore, the oral infection of larval fish through live feed has been demonstrated [17]. Live prey carries a large diversity of associated microbiota that, although they are not pathogenic to live prey, can be transferred to the larval culture, causing detrimental effects [18,19]. The dominant bacterial groups in rotifers are *Vibrio*, *Pseudomonas*, and *Aeromonas*, which account for 10^7^ colony forming units (CFU) per mL in the rearing water or 10^4^–10^5^ CFU per rotifer [8,20,21], with similar bacterial levels observed in *Artemia* [22]. In another study, *Vibrio* was found to be the dominant genus in rotifer cultures, constituting up to 56% of the bacterial community, with *Vibrio anguillarum*, *Vibrio alginolyticus*, *Vibrio diazotrophicus*, *Vibrio mediterranei*, and *Vibrio tubiashii*-like as representative species [8].

Similarly, several investigators have reported that Vibrionaceae are the most common members of the gut flora of feeding larvae [23,24,25]. Grisez et al. [26] reported that the incidence of non-*Vibrio* taxa during the first weeks of larval development was as high as the incidence of vibrios because the digestive tract was not yet sufficiently developed to produce selective conditions.

The importance of bacterial content in the live feed used to feed marine fish larvae under rearing conditions has prompted the need for the development of various strategies to improve the sanitary quality of live feed [27,28,29]. Different bacterial control measures, such as chemical and physical disinfection, use of antibiotics, and the addition of microalgae or probiotics have been studied [30,31,32,33,34,35,36]. In various studies, probiotic strains were proven to be highly effective against pathogens and/or significantly enhance the growth of rotifers and fish larvae [37,38,39,40,41,42].

It must be noted that the use of antibiotics to treat live feed used in fish larval culture is commonplace but their efficacy is clearly species-specific and variable. Additionally, the selection of drug-resistant bacteria in treated live feed has been reported previously [43].

The use of antibiotics as prophylactic agents to control bacterial contaminants in live feed is a routine practice in Chilean larval aquaculture. Among the antibiotics currently approved for use in Chilean aquaculture, florfenicol was by far the most frequently used during 2016, accounting for 82.5% of the antibiotics used in salmon farming [44], and is currently the most used antibacterial agent in marine larval cultures. Due to the intensive and widespread use of this antibiotic, florfenicol resistance has increased in aquaculture settings [45,46,47]. Furthermore, the observation that many of the genes encoding for phenicol resistance reside on mobile genetic elements, including plasmids [46,48], which usually carry other resistance determinants [46,48], the co-selection and persistence of these resistance genes may occur through the use of other antimicrobial agents. In addition, other mechanisms of gene transfer, besides plasmid transfer, in the dissemination of antibacterial resistance among bacteria from fish farm environments have been suggested [49]. Knowledge of the prevalence of antibacterial-resistant bacteria and associated antibiotic-resistance genes (ARGs) is essential for the proper design of control strategies to improve the sanitary quality of live feed and to reduce the risk of introducing high levels of antibacterial-resistant bacteria, which could act as opportunistic pathogens for reared fish larvae. However, studies on the occurrence of antibacterial resistance among bacteria carried by live-feed cultures are still very scarce.

To our knowledge, no studies have previously been performed on the bacterial content of live feed used to feed reared marine fish larvae in Chile, including red cusk eel *G. chilensis*, which is currently considered a priority for Chilean aquaculture. Thus, this study will add important information on the sanitary status of live feeds and the impact of using florfenicol on the bacterial content of live-feed cultures. It could be concluded that medication of administered live feed severely increased the levels of antibiotic-resistant bacteria and related ARGs in rotifer and *Artemia* cultures, resulting in an important risk to reared fish larvae.

The main aim of the study was to evaluate the role of unmedicated and florfenicol-medicated live feed administered in a commercial larval culture of red cusk eel *G. chilensis* as an important source of antibacterial-resistant bacteria, along with the main genes encoding for florfenicol-resistance.

## 2. Materials and Methods

### 2.1. Ethics Statement

The study does not need ethical approval.

### 2.2. Live-Feed Culture Sampling

**Figure 1:**
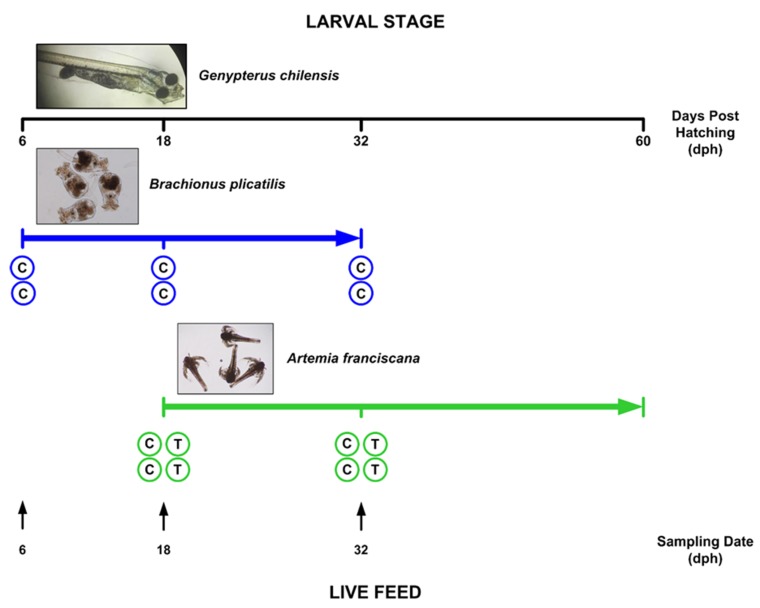
Chronology of the live-feed feeding regime during the larval stage of reared *Genypterus chilensis* and the sampling of live feeds during larval culture. Circles represent live-feed culture tanks deployed in the commercial hatchery. C: untreated cultures; T: florfenicol-treated cultures (20 mg/L). Arrows show the sampling times (dph).

A commercial hatchery was included in the study for the culture of red cusk eel, *G. chilensis*, belonging to Colorado Chile S.A., located approximately 5 km south of Coquimbo at the southern end of La Herradura Bay on the Pacific coast of Chile. The experimental units deployed in the commercial hatchery consisted of two untreated tanks (rotifer *B. plicatilis* and brine shrimp *A. franciscana* nauplii), and additionally duplicated tanks of *A. franciscana* cultures treated once a day with florfenicol (final concentration of 20 mg/L). The length of the larval stage of the reared *G. chilensis* raised with live feed organisms was approximately 60 days post hatching (dph) (Figure 1). Live-feed samples were collected from duplicated culture tanks (600 L at a concentration of 200,000 rotifers per L and 10 L at a concentration of 1,000,000 naupli per L for the rotifer and *Artemia* cultures, respectively) immediately before feeding the reared fish larvae. Samples were taken at 6, 18, and 32 dph during the growth phase in which the larvae were fed with live feed and before feeding with artificial diets (Figure 1). 

For comparative purposes, rotifer and *Artemia* cultures were collected from massive cultures developed in the commercial hatchery. Duplicated cultures were treated with florfenicol (final concentration of 20 mg/L for 2 h), whereas other duplicated untreated cultures were used as untreated controls. After 2 h of exposure, samples from the untreated and florfenicol-treated cultures were collected, placed on ice, immediately transported to the laboratory, and processed within 1 h after collection.

### 2.3. Sample Processing and Culturable Bacterial Count

Live feed cultures were retained on a 65-µm mesh, aseptically concentrated and collected using sterile Falcon tubes (approximately a volume of five mL of concentrated live prey), centrifuged at 4700× *g* for 10 min, and the supernatants were eliminated with a micropipette. The pellets obtained were aseptically weighed and ground by hand using a sterile glass digester with 1 mL of sterile physiological saline (0.85%) (PS) added to obtain a homogenate. Appropriate 10-fold dilutions of the homogenates in PS were prepared and 0.1-mL aliquots were inoculated in triplicate onto agar plates. Culturable counts of heterotrophic and florfenicol-resistant bacteria were determined using a spread plate method with Plate Count Agar (PCA, Becton Dickinson, Sparks, MD, USA), with 2% NaCl added, whereas *Vibrio* spp. counts were determined using Thiosulfate-Citrate-Bile Salts Sucrose agar (TCBS, Becton Dickinson, Sparks, MD, USA) prepared using 50% microfiltered (0.22 µm) aged seawater [50]. Plates with added florfenicol (30 µg/mL, Sigma-Aldrich^®^ (St. Louis, MD, USA) were used to determine the florfenicol-resistant bacteria [50,51]. All plates were incubated at 22 °C for 5 days and the bacterial numbers per gram of sample were calculated as described in Miranda and Rojas [51].

### 2.4. Bacterial Isolates

A total of 65 florfenicol-resistant Gram-negative bacilli were recovered from live feed (27 and 38 isolates from rotifer and *Artemia* cultures, respectively) during the assay as well as from various cultures routinely developed in the hatchery during the period 2018–2019. Isolates were recovered using plates with Tryptic soy agar (TSA, Becton Dickinson, Sparks, MD, USA) containing florfenicol (30 μg/mL) and incubated at 22 °C for 5 days. Isolates were stored at −85 °C in CryoBank^TM^ vials (Mast Diagnostica, Reinfeld, Germany) and were grown in Trypticase soy agar (TSA) at 22 °C for 24 h prior to use.

### 2.5. Bacterial Identification

The Gram staining, cell morphology, oxidase production, oxidation/fermentation (O/F) of glucose, growth on thiosulfate-citrate-bile salts sucrose (TCBS) agar, and susceptibility to the vibriostatic agent O/129 (2,4-diamino-6,7-diisopropylpteridine) (10 and 150 µg per disc) phenotypic tests were conducted according to the procedures described in Buller [52] using media supplemented with NaCl (2%). Furthermore, antimicrobial-resistant isolates were identified using bacterial 16S rRNA gene sequence analysis. For amplification of the 16S rRNA genes, isolates were cultured in Tryptic soy broth (Oxoid, Hants, UK) at 22 °C for 12–24 h and centrifuged at 9000× *g* for 3 min using an Eppendorf 5415D (Eppendorf AG, Hamburg, Germany) microcentrifuge to obtain a pellet. DNA extraction was carried out using the Wizard^®^ Genomic DNA Purification commercial kit (Promega, Madison, WI, USA) following the supplier’s instructions, and the obtained DNA samples were stored at −20 °C until analysis. The amplification of the 16S ribosomal genes of the isolates was carried out using PCR, following the methodology described by Opazo et al. [53]. The resulting amplified PCR products were sequenced with Macrogen (Rockville, MD, USA) using the ABI PRISM 373 DNA Sequencer (Applied Biosystems, Foster City, CA, USA). The sequences were edited and matched to the Ribosomal Database Project [54] to identify the bacterial isolates.

### 2.6. Minimum Inhibitory Concentrations (MICs) of Florfenicol

Minimum inhibitory concentrations (MICs) of florfenicol against all isolates were determined using an agar dilution method, as recommended by the Clinical and Laboratory Standards Institute (CLSI) guideline M07-A10 [55]. A serial two-fold dilution pattern of the antibiotic was added into Mueller–Hinton agar (MH, Becton Dickinson, Sparks, MD, USA) supplemented with NaCl (2%) to obtain final concentrations ranging from 0.5 to 2048 μg/mL. Bacterial suspensions were prepared in sterile 0.85% saline and triplicate plates were inoculated using a Steers replicator apparatus [56], delivering approximately 10^4^ colony forming units per spot, and then incubated for 48 h at 22 °C. The bacterial inocula were applied simultaneously to the agar surfaces using the inoculum-replicating apparatus capable of transferring 32 isolates onto the surface of each plate [55]. The first and last agar plates did not contain any antibiotic in order to detect possible contamination of the isolates or antibiotic carryover. The MIC was defined as the lowest concentration of the antibacterial agent that produced an absence of growth in at least two of the three plates after 48 h. A reference of strain *Escherichia coli* ATCC 25922, recommended by CLSI [57], was used as a quality control organism for verification of the MIC ranges on used Mueller–Hinton agar plates.

### 2.7. Antibacterial Resistance Patterns

The susceptibility of isolates to various antimicrobials was determined using a disk diffusion test according to the Clinical and Laboratory Standards Institute (CLSI) guideline VET3-A [57]. Briefly, the bacterial isolates were resuspended in phosphate-buffered saline (PBS) to obtain a turbidity corresponding to 0.5 McFarland standard (bioMerieux, Marcy-l’Etoile, France), corresponding to a concentration of (1–2) × 10^8^ CFU per mL [55]. Bacterial suspensions were streaked onto plates containing Mueller–Hinton agar (MH) with 2% NaCl added, and disks (Oxoid, Basingstoke, Hampshire, England) containing the following antibiotics were used: amoxicillin (AML, 25 µg), streptomycin (S, 10 μg), kanamycin (K, 30 μg), oxytetracycline (OT, 30 μg), florfenicol (FFC, 30 μg), oxolinic acid (OA, 2 μg), flumequine (UB, 30 μg), and sulfamethoxazole trimethoprim (SXT, 25 μg). Plates were incubated at 22 °C for 24 h according to CLSI guidelines [57], and isolates were considered resistant according to the criteria established by the CLSI [58] or by Miranda and Rojas [51]. *Escherichia coli* ATCC 25922 was used as a quality control strain, as recommended by the CLSI [59]. Several isolates (20%) were re-examined to check the reproducibility of the assay. Furthermore, the antibacterial resistance index (ARI) of untreated and florfenicol-treated larvae cultures was determined according to Hinton et al. [60] using the formula ARI = y/nx, in which y was the actual number of resistance determinants recorded in a population of size n, and x was the total number of antibacterials tested for in the sensitivity test.

### 2.8. Detection of Genes Encoding for Florfenicol Resistance

All isolates were assayed for the presence of genes encoding for florfenicol resistance. The presence of the *floR* and *fexA* genes, encoding for efflux pumps, were detected using the methodology described by Fernández-Alarcón et al. [45] and Higuera-Llantén et al. [61], respectively. Primers F: 5’-AATCACGGGCCACGCTGTATC-3′ and R: 5′-CGCCGTCATTCTTCACCTTC-3′ (*floR*) and F: 5’-TTTCGCTGTTCTTGTGTTCG-3′ and R: 5’-ACCTTGGAAAATCCCCATTC-3′ (*fexA*), were used. The amplification conditions were as follows: for the *floR* gene, denaturation at 95 °C for 3 min; 30 cycles of denaturation at 95 °C for 30 sec, annealing at 56 °C for 30 sec, and elongation at 72 °C for 30 sec; and finally, extension at 72 °C for 7 min. For the *fexA* gene, denaturation at 95 °C for 3 min; 45 cycles of denaturation at 95 °C for 30 sec, annealing at 56 °C for 30 sec, and elongation at 72 °C for 30 sec; and finally, extension 72 °C for 7 min. Amplicon sizes of the *floR* and *fexA* genes were 200 and 450 bp, respectively. The amplified PCR products were sequenced using Macrogen (Rockville, MD, USA), and genes were identified using a computational analysis of the Basic Local Alignment Search Tool (BLAST) sequence alignment against the gene sequences included in the GenBank nucleotide sequence database. For the detection of *floR* and *fexA* genes, strain *Citrobacter freundii* FB98 carrying the *floR* gene [45] and a sequence of gen MH747503.1 [61], flanked by primers *fex*AF and *fex*AR synthesized using Integrated DNA Technologies (IDT, Coralville, IA, USA) were used as positive controls and were included in each PCR run.

### 2.9. Isolation of Plasmid DNA

Bacterial isolates were screened for their plasmid content, as previously described [62]. Briefly, the geneJET Plasmid miniprep kit (Thermo-Scientific, Waltham, MA, USA) was used and the obtained plasmid DNA was run on 1.5% agarose gel electrophoresis for plasmids less than 20 kb and 0.8% agarose gel for plasmids greater than 20 kb. Gels were stained with GelRed^TM^ (Biotium, Hayward, CA, USA) and viewed using UV transillumination. The size was estimated via comparison with known plasmid weight standards and standard molecular weight markers.

### 2.10. Statistical Analysis

Culturable count values were transformed to log_10_, whereas proportions of florfenicol resistance were computed for each live feed (rotifers and *Artemia*) culture and were arcsine-transformed before analysis. One way analysis of variance (ANOVA) [63] was performed to detect significant differences (*p* < 0.05) between culturable counts of heterotrophic and florfenicol-resistant bacteria from control and treated live-feed cultures. Differences between untreated and florfenicol-treated cultures were detected using Student’s *t*-test for two independent samples [63]. In addition, all untreated and treated culture tanks were compared in a nested ANOVA analysis. When differences were statistically significant, the control and treated live feed were then compared against each other using Tukey’s test [63]. The frequencies of resistance to the assayed antimicrobials of the selected isolates were compared with Pearson’s chi-square test, adjusted with Bonferroni’s correction, and *p* < 0.05 was considered to indicate statistical significance. All statistical analyses were carried out using the SPSS version 12.0 computer program [64].

## 3. Results

### 3.1. Culturable Bacterial Counts

According to the current hatchery protocols, rotifer cultures are not treated with antibiotics, in contrast to the *Artemia* cultures, which are routinely treated with florfenicol or other antibacterial agents, thus no treated rotifer mass cultures at the red cusk eel hatchery were available during the sampling period (Table 1).

Florfenicol-resistant bacterial counts were high in both live-feed cultures during the larval growth period, ranging from 10^4^ to 10^7^ CFU/g of rotifer and from 10^6^ to 10^8^ CFU/g of *Artemia* (Table 1). When the florfenicol-resistant bacterial counts from both live-feed cultures were compared, levels of resistant bacteria from *Artemia* cultures were higher than those from rotifer cultures.

When the untreated and florfenicol-treated *Artemia* cultures administered after 18 and 32 days post hatching (dph) of the larval culture were compared, no important differences in heterotrophic bacterial counts were observed (Table 1). By contrast, at 18 and 32 dph of larval culture, the culturable counts of florfenicol-resistant bacteria and *Vibrio* spp. from florfenicol-treated *Artemia* cultures were significantly higher than those from the untreated *Artemia* cultures (Table 1). Furthermore, florfenicol-resistant bacterial counts from untreated and florfenicol treated *Artemia* cultures at 32 dph were higher than those from 18 dph.

Culturable counts of heterotrophic bacteria, florfenicol-resistant bacteria, and *Vibrio* spp. from rotifer cultures at 18 dph were lower than those from the cultures at 6 and 32 dph. Rotifer cultures exhibited their highest culturable counts of heterotrophic bacteria at 32 dph, whereas the highest counts of *Vibrio* spp. and florfenicol-resistant bacteria were observed at 6 and 32 dph. No important differences in the levels of culturable counts of *Vibrio* spp. between 6 and 32 dph were observed.

**Figure 2:**
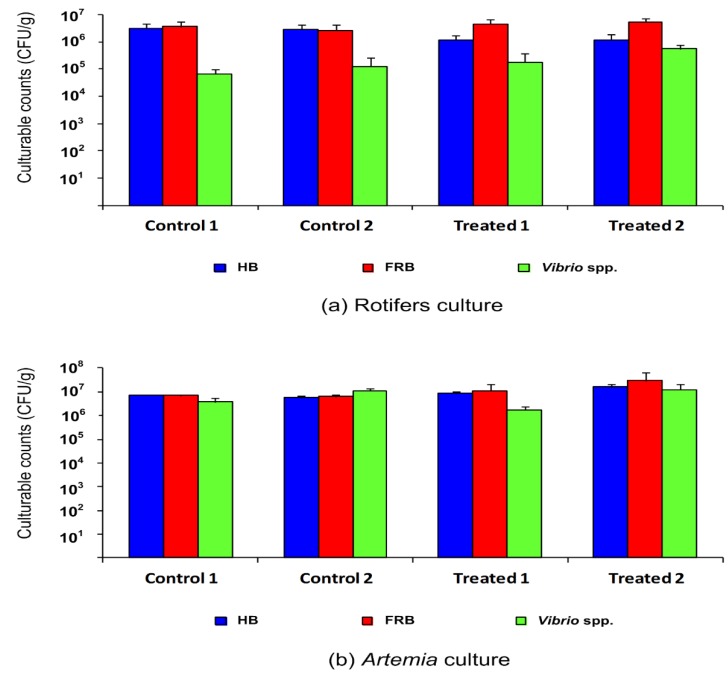
Culturable counts of heterotrophic bacteria (HB), florfenicol-resistant bacteria (FRB), and *Vibrio* spp. from untreated (control) and treated with florfenicol (20 mg/L) rotifer (**a**) and *Artemia* (**b**) cultures. * Significant difference (*p* < 0.05) between untreated and florfenicol-treated cultures.

When rotifer and *Artemia* cultures were treated with florfenicol, bacterial culturable counts remained at high levels (10^5^ to 10^7^ CFU/g), indicating that florfenicol did not reduce the bacterial content in either of the live feeds, as displayed in Figure 2.

Culturable heterotrophic counts from untreated rotifer cultures were significantly higher (*p* = 0.007, Tukey’s test) than those observed in the florfenicol-treated cultures. On the other hand, *Vibrio* spp. counts from untreated rotifer samples ranged from 6.80 × 10^4^ to 1.26 × 10^5^ CFU/g and were significantly lower (*p* = 0.007, Tukey’s test) than those observed in treated rotifer culture samples (from 1.79 × 10^5^ to 5.44 × 10^5^ CFU/g). Otherwise, no significant differences (*p* = 0.065, Tukey’s test) between florfenicol-resistant bacterial counts from untreated and antibiotic-treated rotifer cultures were observed (2.66 × 10^6^ to 3.69 × 10^6^ CFU/g and 4.60 × 10^6^ to 5.33 × 10^6^ CFU/g, respectively) (Figure 2).

Untreated *Artemia* cultures exhibited high levels of heterotrophic bacteria, but significantly lower (*p* = 0.002, Tukey’s test) than those of the antibiotic-treated cultures, whereas the levels of *Vibrio* spp. in untreated and florfenicol-treated cultures were not significantly different (*p* = 0.217, Tukey’s test) (3.67 × 10^6^ to 1.11 × 10^7^ CFU/g and 1.72 × 10^6^ to 1.22 × 10^7^ CFU/g, respectively). The levels of bacteria resistant to florfenicol in samples from treated *Artemia* cultures were high, ranging from 1.14 × 10^7^ to 2.90 × 10^7^ CFU/g, and were significantly higher (*p* = 0.012, Tukey’s test) than those obtained from untreated *Artemia* cultures (6.36 × 10^6^ to 7.09 × 10^6^ CFU/g) (Figure 2).

### 3.2. Bacterial Isolates

The identification of 65 florfenicol-resistant isolates recovered from rotifer and *Artemia* cultures (27 and 38 isolates, respectively) used in the rearing of *G. chilensis* larvae showed a high predominance of *Vibrio* species in both rotifer and *Artemia* cultures (85.2% and 78.9%, respectively). In contrast, a low frequency of isolates belonging to the *Pseudoalteromonas* genus was found in both live-feed cultures (14.8% and 15.8%, respectively) (Table 2; Table 3).

The isolates identified as *Vibrio* spp. via the analysis of their 16S rRNA gene sequences shared the phenotypic properties of the genus *Vibrio*, including Gram-negative, glucose fermenter, oxidase-positive, susceptible to the vibriostatic agent O-129, and able to grow in TCBS agar.

The nucleotide sequences of the isolates were deposited in GenBank under the accession numbers displayed in Table 2 and Table 3.

### 3.3. Minimum Inhibitory Concentrations (MICs)

High levels of florfenicol resistance were observed for the isolates, with MIC values ranging from 32 to 512 µg/mL for isolates recovered from rotifer cultures (Table 4), while the MIC values of isolates from Artemia cultures ranged from 32 to 1024 µg/mL (Table 5). Lower MIC_50_ and MIC_90_ values were exhibited by isolates recovered from rotifer cultures (256 and 256 µg/mL, respectively), whereas higher MIC_50_ and MIC_90_ values (256 and 512 µg/mL, respectively) were observed for isolates from *Artemia* cultures. The reference strain *Escherichia coli* ATCC 25922 used for quality control exhibited an MIC value for florfenicol of 2 µg/mL, which agrees with the values recommended by the National Committee for Clinical Laboratory Standards (NCCLS) [65].

### 3.4. Antimicrobial Resistance Patterns

Similar antimicrobial resistance patterns of the recovered strains were not related to specific bacterial species or the live feed sampled. Furthermore, resistant isolates from untreated and treated cultures exhibited similar antibacterial resistance patterns, as shown in Table 4 and Table 5. Furthermore, similar antimicrobial indexes (ARI) were observed among the florfenicol-resistant bacteria isolated from both live-feed cultures, exhibiting ARI values of 0.79 and 0.71 for isolates recovered from rotifer and *Artemia* cultures, respectively. The majority of isolates from the rotifer and *Artemia* cultures exhibited multiresistance or simultaneous resistance to at least three classes of antimicrobials (24 out of 27 and 36 out of 38 isolates recovered from rotifer and *Artemia* cultures, respectively) (Table 4; Table 5). High levels of multiresistance among florfenicol-resistant isolates from both cultures were detected, with 70.4% of isolates from rotifer cultures exhibiting simultaneous resistance to 7–8 antimicrobials, whereas a high proportion of isolates from *Artemia* cultures were resistant to 5–6 antimicrobials (76.3%). The reference strain *Escherichia coli* ATCC 25922 used for quality control exhibited a zone diameter of inhibition of 23 mm, which agrees with the values recommended by CLSI [57].

**Figure 3:**
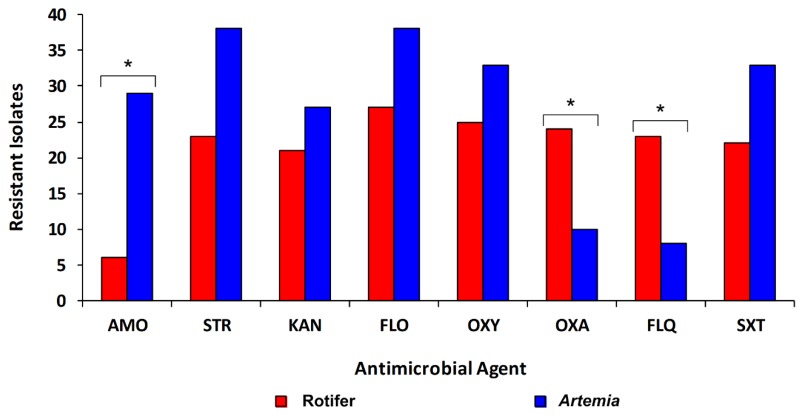
Antibacterial resistance of studied bacterial isolates recovered from rotifer (n = 27) and *Artemia* (n = 38) cultures used to feed *Genypterus chilensis* larvae. AMO: Amoxicillin; STR: Streptomycin; KAN: Kanamycin; FLO: Florfenicol; OXY: Oxytetracycline; OXA: Oxolinic acid; FLQ: Flumequine; SXT: Sulfamethoxazole-trimethoprim. * Significant difference (*p* < 0.05) between rotifer and *Artemia* cultures.

A high proportion of florfenicol-resistant isolates recovered from rotifer and *Artemia* cultures exhibited resistance to the antibacterials streptomycin (93.8%), oxytetracycline (89.2%), co-trimoxazole (84.6%), and kanamycin (73.8%), and did not show significant differences between the cultures (Figure 3). In contrast, significant differences (*p* < 0.05) between isolates from rotifer and *Artemia* cultures for resistance to oxolinic acid (88.2% and 26.1%, respectively), flumequine (85.5% and 21.2%, respectively), and amoxicillin (22.6% and 76.1%, respectively) were detected (Figure 3).

### 3.5. Phenicol Resistance Genes

A high occurrence of the floR gene was detected among the floRfenicol-resistant isolates recovered from rotifer and Artemia cultures when compared with the occurrence of the fexA gene. A high proportion of the resistant isolates (21 out of 27 strains (77.8%)) recovered from the rotifer cultures carried the *floR* gene, and among them, three isolates also carried the *fexA* gene (Table 2). On the other hand, only one isolate carried only the *fexA* gene, whereas in five resistant isolates, none of the assayed genes were detected. Among the isolates recovered from the *Artemia* cultures, 23 out of 38 isolates (60.5%) harbored the *floR* gene, and among them, only one isolate (2.6%) also carried the *fexA* gene (2.6%). In contrast, the *fexA* gene was detected in only five isolates (13.2%), whereas ten isolates (26.3%) from *Artemia* cultures were negative for the assayed genes (Table 3). Resistant isolates carrying neither the *floR* nor *fexA* genes (5 and 10 isolates from rotifer and *Artemia* cultures, respectively) exhibited MIC values of 32 to 128 µg/mL for isolates from rotifer cultures, and MIC values of 32 to 1024 µg/mL for isolates from *Artemia* cultures.

### 3.6. Plasmid Content

Plasmid DNA was detected in 42 out of 59 florfenicol-resistant isolates examined, and from these, 14 and 28 isolates were recovered from rotifer and *Artemia* cultures, respectively (Table 2; Table 3). Interestingly, a higher number of rotifer isolates were negative for plasmid content (13 out of 27 isolates), compared to the isolates recovered from *Artemia* cultures (4 out of 32 assayed isolates). Among the plasmid-carrying isolates, 1 and 11 isolates from rotifer and *Artemia* cultures, respectively, contained two plasmid bands. The number and size of plasmid bands found are shown in Table 2 and Table 3. The majority of resistant isolates from rotifer and *Artemia* cultures contained plasmids with molecular weights ranging from 35 to 50 kb (12 and 29 isolates, respectively). The most commonly found plasmid bands had molecular weights of 40 and 35 kb (16 and 18 isolates) (Table 4; Table 5).

## 4. Discussion

Considering the high importance of florfenicol in treating and preventing bacterial infections in Chilean aquaculture, the occurrence of bacterial resistance to this compound was investigated in live feeds used in larval cultures of red cusk eel, *G. chilensis*, which is considered a priority for Chilean aquaculture. In addition, the presence of the main determinants conferring resistance to florfenicol and the plasmid content of the selected resistant bacteria was investigated.

To our knowledge, this is the first report on the occurrence of antimicrobial-resistant bacteria in the live feeds that are most frequently used in marine fish larvae rearing, as most of the previous studies have attempted to reduce bacterial load in these cultures [19,25,26,27,28,29] or characterize the bacterial species associated with the live feeds [7,8,9,10,11,13,15,16,18,19,43]. Our findings have demonstrated that live-feed cultures used in a Chilean commercial hatchery of red cusk eel have high levels of resistant bacteria in both untreated and treated live feeds, suggesting that the usage of florfenicol is not a necessary causal condition for the development and maintenance of elevated frequencies of florfenicol resistance. That is to say, mass-produced live feeds in the commercial hatchery never exposed to antibacterial therapy exhibited high levels of florfenicol-resistant bacteria. Thus, the well-accepted paradigm that a selective pressure is needed for the selection and persistence of antibacterial resistance in marine fish aquaculture is not supported. Previous findings obtained from a Chilean scallop hatchery, in which high levels of antibacterial resistant bacteria were detected in untreated scallop larvae [66], did support the paradigm.

In commercial hatchery operations, the practice of rotifer batch cultivation characterized by high rotifer densities and high loads of organic matter favors the proliferation of copiotrophic bacteria, such as *Vibrio* spp. Antibacterial treatments were shown to be ineffective at reducing the bacterial load in live feed, and they may even increase the occurrence of vibrios exhibiting antibacterial resistance. In this study, treatments of live-feed cultures with florfenicol were not able to reduce the bacterial load, but most probably led to the emergence of antibiotic-resistant bacteria, thus impeding the establishment of normal non-pathogenic bacteria.

The high levels of florfenicol-resistant bacteria in both live feeds used in marine fish larval culture indicates a widespread antibacterial resistance within the microbiota associated with red cusk eel farming. As was observed, live-feed cultures treated with florfenicol exhibited no significant reductions in their bacterial content and resistant bacteria, and most of the culturable levels observed during the fish larval rearing period remained high (>10^6^ CFU/g). In a previous study, no significant differences (*p* < 0.05) in the culturable counts of heterotrophic bacteria and *Vibrio* spp. between untreated and oxolinic-acid-treated rotifer cultures were observed, resulting in the conclusion that antibiotic-treated rotifers used to feed reared larvae of blue-fin seabream are not needed to improve larval survival [67]. By contrast, the bacterial loads on both rotifers and *Artemia* were reduced with antibiotics [10], but until now, effective bacterial reduction in mass-produced rotifers remains an unsolved issue. Having previously reported that the use of rotifers with reduced bacterial load improved the survival of turbot larvae during first feeding [10], it appears to be highly important to reduce the bacterial load of the rotifer culture prior to use in red cusk eel culture.

However, it is important to consider that variations in several aspects of florfenicol administration in live feeds, such as the purity of antibiotic used, administered concentration by volume, and routes and frequency of administration of florfenicol could preclude the efficacy of antibiotic treatment to significantly reduce bacterial loads in live feeds. To date, there exists only one study of the use of florfenicol that reduced the bacterial load in *Artemia* culture, using a dose of 300 mg/L of florfenicol and resulting in an important reduction of *Vibrio* counts. In this study, when florfenicol was administered, *Vibrio* counts were reduced from 1.20 × 10^9^ ± 9.00 × 10^8^ CFU/g to 2.00 × 10^5^ ± 7.00 × 10^4^ CFU/g and from 4.50 × 10^8^ ± 2.40 × 10^8^ CFU/g to 3.10 × 10^7^ ± 7.00 × 10^6^ CFU/g of *Artemia* nauplii (capsulated and decapsulated cysts, respectively) [68]. However, the potentially pathogenic species, *V. alginolyticus* and *V. parahaemolyticus* remained in the treated *Artemia* naupli [68]. *Vibrio* counts of treated *Artemia* observed in this study were similar to those observed in *Artemia* nauplii (decapsulated cysts), but lower than those observed in capsulated cysts in the previous study; however, it must be noted that in this study, a much lower concentration of florfenicol was administered (20 mg/L versus 300 mg/L).

Despite the previous observations, antibiotic treatment can reduce bacterial load in live feeds but cannot prevent the live feeds from being recolonized within a short period [27], thus suggesting the need to find other therapeutic alternatives. In addition, well-documented toxic effects of florfenicol on fish and crustacean larvae [69,70,71,72] indicate the urgent need for considering the use of alternative strategies to control pathogenic bacteria in live feeds administered to marine fish aquaculture, including bacteriophages, probiotics, and natural products [42,73,74]. In a recent study, probiotics, organic acids, and essential oils were demonstrated to be effective at controlling the pathogenic activity of vibrios on *A. franciscana* nauplii [75]. Thus, using live feeds as carriers for non-antibiotic therapeutic applications in marine fish cultures appears to be a very attractive option.

It has been argued that live feeds carry a low diversity of associated antibiotic-resistant microbiota, composed mostly of vibrios, which are most probably transferred to the fish larval culture, causing detrimental effects on the reared larvae [10,11]. Although no information on florfenicol-resistant species associated with live feeds used in marine fish larval culture is currently available, the dominance of *Vibrio* observed in this study are in accordance with other studies showing *Vibrio* species as the dominant component of the microbiota in the routine production of the rotifer *Brachionus plicatilis* [8,76]. Furthermore, in a previous study, *Vibrio* spp. were reported as the main constituents of the bacterial microbiota in the gut of the turbot larvae, and the authors concluded they were probably introduced by the rotifers [11].

Although phylogenetic analysis based on 16S rRNA gene sequences confirmed that 53 out of 65 isolates belonged to the genus *Vibrio*, 16S rRNA sequence analysis is unable to resolve closely related species, such as those clustered in the *Vibrio* group [77]. In this study, most of the studied isolates identified as *Vibrio* spp. (41 out of 53 isolates) exhibited low percentages of identity (≤99.8%). Thus, resistant isolates were identified only to the genus level, and further analysis, such as multilocus sequence analysis (MLSA) using various housekeeping genes or whole-genome sequencing (WGS), is required to accurately define the taxonomic affiliations of *Vibrio* isolates, especially at the species level [78,79].

This study demonstrated that live feed is a main source of antibiotic-resistant *Vibrio* in the red cusk eel culture, which is a highly significant risk to the health status of reared fish larvae fed with rotifer and *Artemia* considering that previous studies have demonstrated the pathogenic role of various *Vibrio* species in the experimental culture of this species in Chile [3,4,5]. However, further studies are required to elucidate the impact on the microbiota of the larvae reared using these live feeds.

To our knowledge, no studies of resistant microbiota and the associated antibiotic-resistance genes (ARGs) of reared marine fish in Chile have been undertaken despite the intensive use of antibiotics in this industry, which are mainly used in the larval culture and the role of live feeds as carriers of bacteria to reared fish larvae. However, the prevalence and concentration of ARGs, as well as their sources and spreading pathways in marine aquaculture, are little understood. Thus, the incidence of antibacterial resistance and the characterization of related genes is an urgent necessity for this industry, but information on the occurrence of resistance genes in farmed marine fish species in Chile is still very scarce.

As was previously described [46], bacterial resistance to florfenicol is primarily mediated by the *floR* gene, which is a specific drug exporter that confers resistance to florfenicol and chloramphenicol [80], and is widely disseminated among Gram-negative bacteria from animal agricultural sources [81,82,83]. The presence of *floR* has previously been reported in bacteria isolated from mucus and intestinal contents of farmed salmon, as well as lake water and sediments associated with salmon farming in Chile [45,61]. However, this is the first report of the presence of *floR* and *fexA* genes in non-salmonid marine fish aquaculture in Chile. It is interesting to note the high incidence of the *floR* gene among isolates recovered from both live-feed cultures (67.7%) is in accordance with isolates recovered from freshwater and marine environments impacted by salmon farms in Chile [45,61]. Furthermore, this is the first report of *floR*- and *fexA*-carrying *Vibrio* spp. isolated from live feed used in marine fish larval culture, prompting the necessity of implementing continuous monitoring and sanitary control of live feeds to avoid the spread of these resistance genes in the hatchery.

Furthermore, in a previous study, the *fexA* gene was detected in a high number of bacteria, which were mainly identified as *Pseudomonas* and were recovered from the fecal content of salmon reared in a Chilean marine farm that made intensive use of antibacterials [61], contrasting with the low occurrence of *fexA*-carrying isolates observed in this study. These authors concluded that the gut microbiota of medicated farmed salmon could serve as a perfect reservoir for genes encoding for resistance to florfenicol, such as *floR* and *fexA* [61], but that these genes had not thus far been reported in non-salmonid farmed species.

From this study, a high number of isolates exhibited a co-resistance to oxytetracycline, the second-most frequently used antibacterial in Chilean aquaculture. This concurs with previous studies that have reported an association of the *floR* gene with tetracycline resistance. Dang et al. [84] detected the *floR* gene associated with several tetracycline-resistant isolates from aquaculture in China, whereas Gordon et al. [83] found that the *floR* gene was linked to a tetracycline resistance gene in an *Aeromonas bestiarum* isolate recovered from a freshwater environment. This fact might preclude the efficacy of the therapeutic use of oxytetracycline in marine fish larval culture.

Considering that none of the assayed resistance genes were detected in 39.5% of the studied florfenicol-resistant isolates, we could conclude that other mechanisms, such as enzymes or other exporter systems, could be responsible for the exhibited resistance; however, further research is required to elucidate the mechanisms conferring the florfenicol resistance detected in these isolates.

Finally, florfenicol-resistance-encoding genes, mainly *floR*, have frequently been associated with plasmids carried by human and animal pathogenic bacteria [85,86,87,88,89], thus the co-presence of this gene and plasmid elements in a high number of the studied isolates (71.2%) provides very favorable conditions for the genetic exchange among bacteria comprising the live feed and fish larval gut microbiota. This increases the feasibility of the spread of florfenicol-encoding genes to bacterial pathogens. However, the ability of detected plasmids and florfenicol-encoding genes to be transferred horizontally was not addressed in this study and remains to be investigated to properly determine the potential risk of transfer of these elements to the fish larvae microbiota.

Thus, it is critical for the marine fish aquaculture industry in Chile to monitor the emergence and spread of resistance to florfenicol and the associated main components of the resistome and mobilome to avoid future therapy failures in marine fish larval cultures.

## 5. Conclusions

The results of this study have demonstrated that live feed administered to reared larvae played an important role as a major source of antimicrobial-resistant bacteria and associated determinants in the commercial culture of red cusk eel larvae. In addition, the study revealed that florfenicol-resistant microbiota from live feed administered to reared fish larvae mainly belonged to the *Vibrio* and *Pseudoalteromonas* genera and that they exhibited co-resistance to oxytetracycline, streptomycin, and co-trimoxazole. Furthermore, the high prevalence of plasmids among *floR*-carrying bacteria suggests that these bacteria may contribute to the increased spread of bacterial resistance to florfenicol and other antibacterials in environments associated with these cultures.

## Figures and Tables

**Table 1:** Heterotrophic bacteria (HB), florfenicol-resistant bacteria (FRB), and *Vibrio* spp. in mass live-feed cultures used for rearing *G. chilensis* larvae.

Day	Culture	Group	Culturable Counts ± SD (CFU/g)			
			Control Cultures		Treated Cultures	
			1	2	1	2
6	Rotifer	HB	7.90 × 10^8^ ± 9.16 × 10^7^	5.80 × 10^8^ ± 2.33 × 10^8^	N/A	N/A
		FRB	1.03 × 10^7^ ± 4.94 × 10^6^	8.29 × 10^6^ ± 2.39 × 10^6^	N/A	N/A
		*Vibrio*	3.04 × 10^8^ ± 4.48 × 10^7^	1.48 × 10^8^ ± 4.95 × 10^7^	N/A	N/A
18	Rotifer	HB	2.21 × 10^8^ ± 2.11 × 10^8^	2.57 × 10^8^ ± 1.37 × 10^8^	N/A	N/A
		FRB	8.03 × 10^4^ ± 1.04 × 10^4^	5.20 × 10^5^ ± 3.74 × 10^5^	N/A	N/A
		*Vibrio*	3.29 × 10^7^ ± 9.05 × 10^6^	3.08 × 10^7^ ± 2.08 × 10^7^	N/A	N/A
18	*Artemia*	HB	4.21 × 10^8^ ± 1.68 × 10^8^	3.09 × 10^8^ ± 2.37 × 10^8^	3.81 × 10^8^ ± 2.45 × 10^8^	1.58 × 10^8^ ± 5.21 × 10^7^
		FRB	1.57 × 10^7^ ± 7.88 × 10^6^	1.47 × 10^6^ ± 4.88 × 10^4^	3.82 × 10^7^ ± 1.58 × 10^7^	7.84 × 10^7^ ± 1.47 × 10^7^
		*Vibrio*	1.62 × 10^7^ ± 1.46 × 10^7^	3.60 × 10^4^ ± 2.70 × 10^4^	1.82 × 10^7^ ± 8.44 × 10^6^	4.42 × 10^6^ ± 3.08 × 10^6^
32	Rotifer	HB	6.79 × 10^8^ ± 9.57 × 10^7^	1.71 × 10^9^ ± 2.63 × 10^8^	N/A	N/A
		FRB	1.12 × 10^6^ ± 9.57 × 10^5^	1.79 × 10^7^ ± 7.27 × 10^6^	N/A	N/A
		*Vibrio*	8.33 × 10^7^ ± 3.56 × 10^7^	2.63 × 10^8^ ± 8.25 × 10^7^	N/A	N/A
32	*Artemia*	HB	4.55 × 10^8^ ± 2.98 × 10^8^	1.74 × 10^9^ ± 4.99 × 10^8^	1.27 × 10^9^ ± 3.66 × 10^8^	1.21 × 10^9^ ± 7.55 × 10^8^
		FRB	3.33 × 10^7^ ± 2.01 × 10^7^	1.00 × 10^8^ ± 5.92 × 10^7^	1.22 × 10^8^ ± 7.18 × 10^7^	3.50 × 10^8^ ± 6.41 × 10^7^
		*Vibrio*	1.20 × 10^7^ ± 1.38 × 10^7^	3.54 × 10^8^ ± 1.41 × 10^8^	2.14 × 10^6^ ± 1.05 × 10^7^	4.02 × 10^7^ ± 2.44 × 10^6^

N/A: Not available.

**Table 2:** Identification, source, and phenotypic properties of florfenicol-resistant isolates recovered from rotifer cultures.

Isolate	Source	Phenotypic Properties					Accession No.	Closest Species (% Identity)
		Gram	OF Glucose	Oxidase	O-129	Growth on TCBS		
RVF24	Treated culture	−	F	+	S	+	MN920721	*Vibrio azureus* (100.00)
RVF27	Untreated culture	−	F	+	S	+	MN920722	*Vibrio xuii* (99.65)
RVF33	Treated culture	−	F	+	S	+	MN920723	*Vibrio xuii* (99.49)
RGF51	Untreated culture	−	I	+	R	−	MN920724	*Pseudoalteromonas carrageenovora* (99.49)
RGF65	Treated culture	−	I	+	R	−	MN920725	*Pseudoalteromonas tetraodonis* (90.81)
RGF67	Treated culture	−	I	+	R	−	MN920726	*Pseudoalteromonas paragorgicola* (99.04)
RGF70	Treated culture	−	I	+	R	−	MN920727	*Pseudoalteromonas carrageenovora* (99.15)
RGT71	Untreated culture	−	F	+	S	+	MN920728	*Vibrio alginolyticus* (98.97)
RGFR106	Untreated culture	−	F	+	S	+	MN920729	*Vibrio xuii* (98.56)
RGFR107	Untreated culture	−	F	+	S	+	MN920730	*Vibrio xuii* (98.25)
RGFR108	Untreated culture	−	F	+	S	+	MN955424	*Vibrio xuii* (98.01)
RGFR109	Untreated culture	−	F	+	S	+	MN920731	*Vibrio xuii* (98.60)
RGFR112	Treated culture	−	F	+	S	+	MN920732	*Vibrio xuii* (97.31)
RGFR113	Treated culture	−	F	+	S	+	MN920733	*Vibrio xuii* (97.91)
RGFR114	Treated culture	−	F	+	S	+	MN920734	*Vibrio algoinfesta* (97.08)
RGFR115	Treated culture	−	F	+	S	+	MN920735	*Vibrio xuii* (97.21)
RGFR116	Treated culture	−	F	+	S	+	MN920736	*Vibrio xuii* (97.95)
RGFR118	Treated culture	−	F	+	S	+	MN920737	*Vibrio xuii* (97.80)
RGFR119	Treated culture	−	F	+	S	+	MN920738	*Vibrio xuii* (97.23)
RGFR120	Treated culture	−	F	+	S	+	MN920739	*Vibrio xuii* (98.08)
RGFR121	Treated culture	−	F	+	S	+	MN920740	*Vibrio xuii* (97.65)
RGTR139	Untreated culture	−	F	+	S	+	MN920741	*Vibrio xuii* (99.36)
RGTR146	Untreated culture	−	F	+	S	+	MN920742	*Vibrio xuii* (99.48)
RGTR150	Treated culture	−	F	+	S	+	MN920743	*Vibrio algoinfesta* (99.78)
RGTR154	Treated culture	−	F	+	S	+	MN920744	*Vibrio xuii* (98.45)
RGTR157	Treated culture	−	F	+	S	+	MN920745	*Vibrio xuii* (98.55)
RGTR160	Treated culture	−	F	+	S	+	MN920746	*Vibrio xuii* (99.54)

OF: Oxidation/Fermentation; O-129: 2,4-diamino-6,7-diisopropylpteridine; TCBS: Thiosulphate Citrate Bile Salt Agar; F: Fermenter; S: Susceptible; R: Resistant.

**Table 3:** Identification, source, and phenotypic properties of florfenicol-resistant isolates recovered from *Artemia* cultures.

Isolate	Source	Phenotypic Properties					Accession No.	Closest Species (% Identity)
		Gram	OF Glucose	Oxidase	O-129	Growth on TCBS		
AVF09	Untreated culture	−	F	+	S	+	MN920747	*Vibrio tasmaniensis* (99.57)
AVF32	Untreated culture	−	F	+	S	+	MN920748	*Vibrio alginolyticus* (99.53)
AVF45	Untreated culture	−	F	+	S	+	MN920749	*Vibrio toranzoniae* (99.72)
AVF53	Untreated culture	−	F	+	S	+	MN920750	*Vibrio rotiferianus* (95.90)
AVF58	Untreated culture	−	F	+	S	+	MN920751	*Vibrio rotiferianus* (95.90)
AVF60	Untreated culture	−	F	+	S	+	MN920752	*Vibrio neocaledonicus* (100.00)
AVF61	Untreated culture	−	F	+	S	+	MN920753	*Vibrio harveyi* (100.00)
AVF68	Untreated culture	−	F	+	S	+	MN920754	*Vibrio toranzoniae* (99.14)
AGF75	Untreated culture	−	F	+	S	+	MN920755	*Vibrio natriegens* (98.35)
AGF78	Untreated culture	−	O	+	R	−	MN920756	*Pseudomonas pachastrellae* (98.14)
AGF82	Untreated culture	−	F	+	S	+	MN920758	*Vibrio alginolyticus* (100.00)
AGF83	Untreated culture	−	F	+	S	+	MN920760	*Vibrio alginolyticus* (99.90)
AGF85	Untreated culture	−	F	+	S	+	MN920773	*Vibrio alginolyticus* (99.89)
AGF86	Untreated culture	−	F	+	S	+	MN920759	*Vibrio neocaledonicus* (97,62)
AGF100	Treated culture	−	F	+	S	+	MN920757	*Vibrio parahaemolyticus* (100.00)
AGF101	Treated culture	−	F	+	S	+	MN920774	*Vibrio natriegens* (100.00)
AGF104	Untreated culture	−	F	+	S	+	MN920775	*Vibrio alginolyticus* (100.00)
AGF105	Untreated culture	−	F	+	S	+	MN920776	*Vibrio natriegens* (99.69)
AGT90	Untreated culture	−	O	+	R	−	MN920761	*Pseudoalteromonas ganghwensis* (99.69)
AGT92	Untreated culture	−	O	+	R	−	MN920777	*Pseudoalteromonas tetraodonis* (94.08)
AGT93	Untreated culture	−	O	+	R	−	MN920766	*Psychrobacter pacificensis* (83.80)
AGT94	Untreated culture	−	O	+	R	−	MN920767	*Pseudoalteromonas atlantica* (98.05)
AGT103	Untreated culture	−	F	+	S	+	MN920778	*Vibrio neocaledonicus* (99.90)
AGT104	Untreated culture	−	O	+	R	−	MN920779	*Pseudoaleteromonas carrageenovora* (85.03)
AGT106	Untreated culture	−	I	+	R	−	MN955425	*Pseudoalteromonas shioyasakiensis* (97.20)
AGT109	Treated culture	−	F	+	S	+	MN920780	*Vibrio hyugaensis* (98.30)
AGT110	Treated culture	−	F	+	S	+	MN920768	*Vibrio alginolyticus* (97.24)
AGT111	Treated culture	−	F	+	S	+	MN920783	*Vibrio xuii* (97.64)
AGT112	Treated culture	−	F	+	S	+	MN920765	*Vibrio alginolyticus* (99.80)
AGT114	Treated culture	−	F	+	S	+	MN920764	*Vibrio neocaledonicus* (99.60)
AGT119	Untreated culture	−	F	+	S	+	MN920763	*Vibrio parahaemolyticus* (99.30)
AGT122	Treated culture	−	F	+	S	+	MN920769	*Vibrio azureus* (98.29)
AGT123	Treated culture	−	F	+	S	+	MN920770	*Vibrio diabolicus* (99.90)
AGT124	Treated culture	−	F	+	S	+	MN920771	*Vibrio alginolyticus* (99.79)
AGT125	Treated culture	−	F	+	S	+	MN920772	*Vibrio alginolyticus* (98.65)
AGT127	Treated culture	−	O	+	R	−	MN920781	*Pseudoalteromonas* tetraodonis (86.82)
AGT128	Treated culture	−	F	+	S	+	MN920782	*Vibrio alginolyticus* (98.23)
AGT129	Treated culture	−	F	+	S	+	MN920762	*Vibrio parahaemolyticus* (97.71)

OF: Oxidation/Fermentation; O-129: 2,4-diamino-6,7-diisopropylpteridine; TCBS: Thiosulphate Citrate Bile Salt Agar; F: Fermenter; S: Susceptible; R: Resistant.

**Table 4:** Minimum inhibitory concentrations (MICs) of florfenicol (FLO), antimicrobial resistance patterns, florfenicol-resistance-encoding genes, and plasmid content of resistant isolates recovered from rotifer cultures.

Isolate	MIC FLO (µg/mL)	Resistance Pattern	FLO Resistance Genes		No. of Plasmids	Approximate Size (kb)
			*floR*	*fexA*		
RVF24	64	AMO-STR-KAN-FLO-OXY-OXA-FLQ	−	−	0	−
RVF27	64	AMO-STR-KAN-FLO-OXY-OXA-FLQ-SXT	−	+	0	−
RVF33	32	AMO-STR-KAN-FLO-OXY-OXA-FLQ-SXT	−	−	0	−
RGF51	256	FLO-OXY	+	+	0	−
RGF65	256	FLO-OXY-OXA-FLQ-SXT	+	+	1	1.4
RGF67	256	FLO-OXY	−	−	0	−
RGF70	256	FLO-OXA	−	−	0	−
RGT71	128	AMO-STR-KAN-FLO-OXY	+	−	2	40/60
RGFR106	256	STR-KAN-FLO-OXA-FLQ-SXT	+	−	1	40
RGFR107	256	STR-KAN-FLO-OXY-OXA-FLQ-SXT	+	+	0	−
RGFR108	512	STR-KAN-FLO-OXY-OXA-FLQ-SXT	−	−	0	−
RGFR109	256	STR-KAN-FLO-OXY-OXA-FLQ-SXT	+	−	0	−
RGFR112	256	STR-KAN-FLO-OXY-OXA-FLQ-SXT	+	−	1	40
RGFR113	256	STR-KAN-FLO-OXY-OXA-FLQ-SXT	+	−	0	−
RGFR114	256	STR-KAN-FLO-OXY-OXA-FLQ-SXT	+	−	1	40
RGFR115	256	AMO-STR-KAN-FLO-OXY-OXA-FLQ-SXT	+	−	1	80
RGFR116	256	STR-KAN-FLO-OXY-OXA-FLQ-SXT	+	−	1	40
RGFR118	128	STR-KAN-FLO-OXY-OXA-FLQ-SXT	+	−	1	40
RGFR119	256	AMO-STR-KAN-FLO-OXY-OXA-FLQ-SXT	+	−	0	−
RGFR120	256	STR-KAN-FLO-OXY-OXA-FLQ-SXT	+	−	0	−
RGFR121	512	STR-FLO-OXY-OXA-FLQ-SXT	+	−	1	35
RGTR139	128	STR-FLO-OXY-OXA-FLQ-SXT	+	−	0	−
RGTR146	512	STR-KAN-FLO-OXY-OXA-FLQ-SXT	+	−	1	40
RGTR150	256	STR-KAN-FLO-OXY-OXA-FLQ-SXT	+	−	1	40
RGTR154	256	STR-KAN-FLO-OXY-OXA-FLQ-SXT	+	−	1	40
RGTR157	256	STR-KAN-FLO-OXY-OXA-FLQ-SXT	+	−	1	40
RGTR160	256	STR-KAN-FLO-OXY-OXA-FLQ-SXT	+	−	1	35

AMO: Amoxicillin; STR: Streptomycin; KAN: Kanamycin; FLO: Florfenicol; OXY: Oxytetracycline; OXA: Oxolinic acid; FLQ: Flumequine; SXT: Sulfamethoxazole-trimethoprim.

**Table 5:** Minimum inhibitory concentrations (MICs) of florfenicol (FLO), antimicrobial resistance patterns, florfenicol-resistance-encoding genes, and plasmid content of resistant isolates recovered from *Artemia* cultures.

Isolate	MIC FLO (µg/mL)	Resistance Pattern	FLO Resistance Genes		No. of Plasmids	Approximate Size (kb)
			*floR*	*fexA*		
AVF09	32	AMO-STR-FLO-OXY-OXA-FLQ	−	+	1	40
AVF32	32	STR-FLO	−	−	0	−
AVF45	32	STR-KAN-FLO-OXY	−	−	3	5/6/15
AVF53	32	AMO-STR-KAN-FLO-OXY-SXT	−	−	1	40
AVF58	64	AMO-STR-KAN-FLO-OXY-OXA-FLQ-SXT	+	−	1	40
AVF60	32	AMO-STR-KAN-FLO-OXY-OXA-FLQ-SXT	−	−	1	40
AVF61	64	AMO-STR-KAN-FLO-OXY-OXA-SXT	+	−	1	40
AVF68	512	STR-KAN-FLO-OXY-OXA	−	−	1	40
AGF75	512	AMO-STR-FLO-OXY-SXT	+	−	2	35/100
AGF78	256	STR-KAN-FLO-OXY-OXA-FLQ-SXT	−	+	0	−
AGF82	512	AMO-STR-KAN-FLO-OXY-SXT	+	−	1	35
AGF83	512	AMO-STR-KAN-FLO-OXY-SXT	+	−	1	40
AGF85	512	AMO-STR-KAN-FLO-OXY-SXT	+	−	1	35
AGF86	512	AMO-STR-KAN-FLO-SXT	+	−	1	50
AGF100	256	AMO-STR-KAN-FLO-SXT	+	−	0	−
AGF101	256	AMO-STR-KAN-FLO-SXT	+	−	1	35
AGF104	256	AMO-STR-KAN-FLO-OXA-FLQ-SXT	+	−	1	35
AGF105	1024	AMO-STR-FLO-OXY-SXT	+	−	2	35/100
AGT90	256	STR-KAN-FLO-OXY-SXT	−	−	1	40
AGT92	1024	STR-KAN-FLO-OXY-OXA-FLQ-SXT	−	−	0	−
AGT93	128	AMO-STR-FLO-OXY-SXT	−	+	1	40
AGT94	1024	AMO-STR-KAN-FLO-OXY-SXT	−	−	1	50
AGT103	512	AMO-STR-KAN-FLO-OXY-SXT	+	−	1	35
AGT104	128	STR-FLO-OXY-OXA-FLQ	−	+	1	50
AGT106	256	STR-KAN-FLO-OXY-SXT	−	−	1	50
AGT109	512	AMO-STR-KAN-FLO-OXY-SXT	+	+	2	35/100
AGT110	128	AMO-STR-FLO-OXY-SXT	+	−	2	15/45
AGT111	1024	STR-KAN-FLO-OXY-OXA-FLQ-SXT	−	+	2	1.3/2
AGT112	256	AMO-STR-KAN-FLO-OXY-SXT	+	−	1	55
AGT114	128	AMO-STR-FLO-OXY-SXT	+	−	2	15/45
AGT119	256	AMO-STR-FLO-OXY-SXT	+	−	2	15/45
AGT122	512	AMO-STR-FLO-OXY-SXT	+	−	2	15/45
AGT123	256	AMO-STR-KAN-FLO-OXY-SXT	+	−	2	40/60
AGT124	128	AMO-STR-FLO-OXY-SXT	+	−	2	40/60
AGT125	256	AMO-STR-KAN-FLO-OXY-SXT	+	−	1	50
AGT127	512	AMO-STR-KAN-FLO-OXY-SXT	−	−	2	1/40
AGT128	256	AMO-STR-KAN-FLO-OXY-SXT	+	−	1	35
AGT129	512	AMO-STR-KAN-FLO-OXY-SXT	+	−	1	35

AMO: Amoxicillin; STR: Streptomycin; KAN: Kanamycin; FLO: Florfenicol; OXY: Oxytetracycline; OXA: Oxolinic acid; FLQ: Flumequine; SXT: Sulfamethoxazole-trimethoprim.
